# Editorial: Nutrition and quality of life in the elderly

**DOI:** 10.3389/fnut.2024.1429245

**Published:** 2024-06-04

**Authors:** Daniela Caetano Gonçalves, Gabriela Salim de Castro

**Affiliations:** ^1^Department of Biosciences, Universidade Federal de São Paulo (UNIFESP), Campus Baixada Santista, Santos, Brazil; ^2^Cancer Metabolism Research Group, Departamento de Cirurgia and LIM 26-HC da Faculdade de Medicina da Universidade de São Paulo, São Paulo, Brazil

**Keywords:** elderly, quality of life, nutrition-related metabolic diseases, chronic diseases, sarcopenia, osteoporosis, nutritional status

This Research Topic presents 15 articles that discuss strategies to identify and treat major risk factors related to poor health and suggest interventions aiming to mitigate conditions that affect old people. Sedentary behaviors and imbalanced dietary habits are among the principal contributors to these health challenges.

The risk factors related to diseases and quality of life in the elderly and the tools to identify them were evaluated in several articles included in this Research Topic. Brech et al. carried out a cross-sectional study with Brazilian elderly people over 80 years and verified the association between sociodemographic factors and quality of life with their nutritional status. The systematic review of Golmohammadi et al. verified the association between the inflammatory index of the diet and quality of life in the elderly, indicating a possible association between them.

Yang et al. followed, for 4 years, 2,601 participants with prediabetes to verify the six obesity-related indexes that are evaluated in the study could be associated with prediabetes regression. The authors identified that a low initial body roundness index, waist-to-height ratio, and Chinese visceral adiposity index, as well as a reduction in the triglyceride glucose index, were related to the regression of prediabetes to normoglycemia.

The phase angle—an index obtained from bioimpedance—is normally used as an indicator of mortality in several diseases. Langer et al. investigated the relationship between changes in phase angle and mortality and morbidity concerning cardiovascular and coronary heart disease in an 18-year follow-up study and demonstrated that phase angle can be a reliable tool in this investigation.

The study by Li, Shen et al. explored the impact of malnutrition in 662 oldest-old patients hospitalized due to acute coronary syndrome (ACS). The authors reported that malnutrition evaluated through the geriatric nutritional risk index (GNRI) was common in this population with ACS (206 patients with GNRI ≤ 98). Moreover, GNRI could predict all-cause mortality in the oldest-old patients with ACS.

The article by Hu et al. showed that 19.3% of the 254 patients who underwent total hip arthroplasty (THA) exhibited postoperative delirium (POD). The identified risk factors for POD in this population were neutrophil-to-albumin ratio (NAR), prognostic nutritional index (PNI), systemic inflammation score (SIS), and age. Based on these results, the authors developed a POD predictive nomogram model with a C-index value of this model being 0.869 (95% CI = 0.820–0.918).

Ju et al. evaluated the association between the comprehensive geriatric assessment (CGA) and the nutritional status of 211 elderly patients. The Mini Nutritional Assessment (MNA) questionnaire was used to assess the nutritional status. The quality of daily living was measured through the scores of activities of daily living (ADL) and instrumental activities of daily living (IADL). History of peptic ulcer disease, high scores on the Geriatric Depression Scale (GDS), ADL, and IADL were associated with an increased risk of undernutrition.

Zhang et al. hypothesized that the gut microbiota of elderly people with hypertension may differ between two groups of participants: hypertension elderly and hypertension longevity. The hypertension longevity (with a mean age of 100 years) showed efficient acetate absorption and a microbiota composition characterized by higher abundance of *Bacteroides, Faecalibacterium*, and *Alistipes* and lower abundance of *Klebsiella* and *Streptococcus* compared to the hypertension elderly group (with a mean age of 70.5 years). Therefore, the microbiota may have an impact on longevity, despite high levels of systolic blood pressure. An exploratory study by O'Mahony et al. showed that the effects of a Mediterranean diet on the microbiota and health of the elderly are still poorly understood and recognized by the elderly and healthcare professionals.

The study of Wang et al. showed a relationship between serum vitamin D levels and the concentration of irisin—a myokine related to muscle mass. Lower levels of irisin were associated with sarcopenia in elderly women. Xu et al. aimed to investigate the association between the daily intake of certain minerals and the incidence of cataracts in the elderly American population. They found a strong negative association between selenium intake and the incidence of cataracts in this population.

Liu Z. et al. showed that a high intake of vitamin B_12_ was associated with an increased risk of glaucoma in participants aged 40 years or over from the USA population. The authors used data from the National Health and Nutrition Examination Survey (NHANES) from 2005 to 2008 in which dietary intake was evaluated using two 24-h dietary recalls. In total, 594 participants were included. The authors found a significant association between high intake of B_12_ and glaucoma, while normal or low doses of B_12_ intake showed no significant association.

Li, Zhu et al. proposed a study protocol to investigate the effects of vitamin K_2_ in nocturnal leg cramps (NLC)—a common musculoskeletal disorder that causes sudden contractions of the leg. Menaquinone (180 μg/day) will be offered to participants over 65 years of age for 8 weeks. The study hypothesis is that vitamin K2 decreases muscle contractions, thereby alleviating NLC.

The review of Liu B. et al. discusses lipid and glucose metabolism in senescence. The authors brought together information related to decreased oxidation, increased synthesis of fatty acids, and increased glycolysis in senescent cells. Furthermore, the article discusses how external factors, such as diet, environmental pollution, stress, and temperature, can quicken cellular senescence.

Comprehensive palliative care that included local culture and traditions together with a multidisciplinary team provided longer overall survival when compared to standard oncological care offered to patients with non-small cell lung cancer, as showed by Chen et al.. Moreover, this integrative approach resulted in a higher quality of life, a lower percentage of patients with depressive symptoms, and a better nutritional status.

Assessing potential risks and methods for addressing health challenges that affect the elderly, these investigations offer valuable perspectives for supporting the health of old people. Additionally, studies examining areas such as the gut's microbiota, nutrient levels, and metabolic functions unveil promising paths for optimizing health among the elderly. By prioritizing preventive actions and tailored treatments, there are considerable opportunities to elevate the wellbeing of elderly individuals and foster healthy aging.

## Author contributions

DG: Writing – original draft, Writing – review & editing. GC: Writing – original draft, Writing – review & editing.

